# Constructing episodes of inpatient care: data infrastructure for population-based research

**DOI:** 10.1186/1471-2288-12-133

**Published:** 2012-09-03

**Authors:** Randy Fransoo, Marina Yogendran, Kendiss Olafson, Clare Ramsey, Kari-Lynne McGowan, Allan Garland

**Affiliations:** 1 Department of Community Health Sciences, University of Manitoba, Winnipeg, MB, Canada; 2 Department of Community Medicine, University of Manitoba, Winnipeg, MB, Canada; 3 The Manitoba Centre for Health Policy, University of Manitoba, Winnipeg, MB, Canada

## Abstract

**Background:**

Databases used to study the care of patients in hospitals and Intensive Care Units (ICUs) typically contain a separate entry for each segment of hospital or ICU care. However, it is not uncommon for patients to be transferred between hospitals and/or ICUs, and when transfers occur it is necessary to combine individual entries to accurately reconstruct the complete *episodes* of hospital and ICU care. Failure to do so can lead to erroneous lengths-of-stay, and rates of admissions, readmissions, and death.

**Methods:**

This study used a clinical ICU database and administrative hospital abstracts for the adult population of Manitoba, Canada from 2000–2008. We compared five methods for identifying patient transfers and constructing hospital episodes, and the ICU episodes contained within them. Method 1 ignored transfers. Methods 2–5 considered the time gap between successive entries (≤1 day vs. ≤2 days), with or without use of data fields indicating inter-hospital transfer. For the five methods we compared the resulting number and lengths of hospital and ICU episodes.

**Results:**

During the study period, 48,551 hospital abstracts contained 53,246 ICU records. For Method 1 these were also the number of hospital and ICU episodes, respectively. Methods 2–5 gave remarkably similar results, with transfers included in approximately 25% of ICU-containing hospital episodes, and 10% of ICU episodes. Comparison with Method 1 showed that failure to account for such transfers resulted in overestimating the number of episodes by 7-10%, and underestimating mean or median lengths-of-stay by 9-30%.

**Conclusions:**

In Manitoba is it not uncommon for critically ill patients to be transferred between hospitals and between ICUs. Failure to account for transfers resulted in inaccurate assessment of parameters relevant to researchers, clinicians, and policy-makers. The details of the method used to identify transfers, at least among the variations tested, made relatively little difference. In addition, we showed that these methods for constructing episodes of hospital and ICU care can be implemented in a large, complex dataset.

## Background

Evaluations of hospital care, for health services and epidemiology research, and for quality improvement efforts, often utilize large population-based databases. Similar tools are increasingly being used for evaluation of care of critically ill patients in intensive care units (ICUs), which is an important part of modern health care [1,2], and exists within the envelope of hospital care.

Data for ICU research and quality improvement may come from ICU-specific databases, administrative hospital data (hospital abstracts), or both sources. An important feature of such work is the appropriate identification of episodes of hospital and ICU care.

Conceptually, the total contiguous period spent in one or more hospitals after initial entry to hospital represents a single episode of hospital care. If a patient is transferred from one hospital to another the hospital episode includes time in multiple hospitals. Since individual hospital abstracts typically only cover the time spent in a single hospital, accurate creation of hospital episodes including inter-hospital transfer requires identification and merging of multiple hospital abstracts.

The situation for episodes of ICU care is similar. A single episode of ICU care can involve inter-ICU transfers between ICUs in different hospitals, and/or between distinct ICUs in a single hospital (e.g. surgical and medical); however, ICU records typically only cover the time spent in a single ICU. Thus, correct identification of ICU episodes requires identification and merging of multiple ICU records. The difficulty of identifying these distinct episodes of care is magnified by the variety of possible permutations of ICU episodes within hospital episodes; a single hospital episode can include one or more ICU episodes, which themselves can include time in one or more ICUs, in one or more hospitals.

When transfers exist, important errors can result from mistakenly treating individual entries as if they constitute separate, self-contained episodes of care. These include overestimating the number and rates of admission and readmission, and underestimating mortality rates and measures of resource use, such as length-of-stay (LOS). On the other hand, erroneously combining entries that actually represent distinct episodes will have the opposite effects.

In this study we explored different methods of identifying inter-hospital and inter-ICU transfers, using various combinations of admission/discharge timing and locations. For each method, we documented the number of episodes created, and the impact on ICU and hospital LOS.

## Methods

This work was performed at the Manitoba Centre for Health Policy as part of a study on the epidemiology of critical illness in the Canadian province of Manitoba [3]. It was approved by the Health Research Ethics Board of the University of Manitoba, and the Manitoba Health Information Privacy Committee.

All Manitoba residents are covered by a universal, comprehensive health insurance system. Our study included all adult Manitobans who received ICU care anywhere in the province during the eight year period from April 1, 2000 to March 31, 2008. The Manitoba population represents a virtually closed system regarding ICU care, as there are no Canadian hospitals providing ICU care within 150 miles of its borders; consequently all Manitoba residents receive their ICU care in Manitoba, except those needing such care while travelling.

Winnipeg is the capital city of Manitoba, and home to 57% of the provincial population of 1.2 million. The six acute care hospitals in Winnipeg contain 11 adult ICUs of various types, with a total of 82 ICU beds. In addition, the province has 10 other hospitals each with a single ICU, totalling 36 ICU beds.

We used two databases for this study. The hospital abstract database (HADB) includes administrative data maintained by the provincial department of health. Containing information about every admission to all Manitoba hospitals from 1974 onwards, HADB data is collected by abstractors located in each hospital. Abstractors are centrally trained, and use uniform definitions, data collection methods, and data entry software. Each hospital abstract spans the time from entry to separation in a single hospital, so a new hospital abstract is created when a patient is transferred from one hospital to another.

The Winnipeg ICU Database (WICUDB) is a clinical database. It contains detailed information about all adult ICU admissions in Winnipeg hospitals. Each WICUDB record spans the time from entry to separation in a single ICU, so a new record is created when a patient is transferred from one ICU to another. WICUDB data is obtained from bedside medical records by specially trained and dedicated personnel, all of whom are former ICU nurses. This information undergoes extensive testing of the validity of patient identification, and parameter values.

Hospitals in Manitoba occasionally care for non-Manitobans. We identified a person as being a Manitoban by the presence of a valid provincial Personal Health Identification Number (PHIN) in both databases. The current analysis was restricted to Manitoba residents, who comprise 95% of ICU patients in the province [3]. Both databases were de-identified by removal of names and addresses, and replacement of PHINs by a unique scrambled version; for simplicity we will use the term PHIN to refer to these scrambled PHINs.

Two previously described preliminary steps for generating the data infrastructure were required [4]. First, we linked each WICUDB record to a single hospital abstract, with a success rate of 99.2%. Second, we demonstrated that hospital abstracts alone accurately identify the presence and timing of ICU care, enabling us to go beyond the bounds of the Winnipeg ICUs and include the entire province in our analysis.

Constructing episodes of ICU and hospital care from individual hospital abstracts and ICU records requires the ability to identify which ones belong to the same individual. The presence of the PHIN in both databases made this a simple task. The fact that each ICU record was linked to a specific hospital abstract facilitated a two stage, “outside-in” approach of constructing the episodes: first we identified the hospital episodes (outside), and then, to identify ICU episodes contained within a hospital episode (inside) we only had to consider the ICU records linked to the hospital abstracts comprising that hospital episode.

### Identifying episodes of ICU-containing hospital care

This process began with the creation of a list of each resident who had any ICU care during the study period. Then, for each such individual: (a) the PHIN was used to identify all hospital abstracts for that person during the study period, regardless of whether they contained any ICU care, (b) these were arranged chronologically by hospital entry date, and (c) we used Methods 1–5, detailed below, to identify and combine abstracts which were potentially part of the same hospital episode.

Hospital abstract variables used to identify inter-hospital transfers were the timing of hospital entry and separation, and information about the patient’s location before entry and after separation. In referencing two successive hospital abstracts for the same person, we call the earlier-starting one abstract#1 and the later-starting one abstract#2. The “gap” between abstracts was defined as the interval from hospital separation in abstract#1 until hospital entry in abstract#2.

The mandatory hospital abstract field called *SeparationCode* indicates the type of location the patient was discharged to, e.g., home or another hospital. In addition, *HospitalTransferFrom* and *HospitalTransferTo* variables are intended to identify the specific institution the patient came from or went to; unfortunately, these two data fields are optional and not always completed. Anticipating that the timing and location information may sometimes be recorded incorrectly, as with all data, a certain latitude was appropriate in using them to identify inter-hospital transfers.

We assessed differing methods for combining individual hospital abstracts into hospital episodes. Specifically, we examined the impact of: (a) allowing a ≤1-day versus ≤2-day gap between successive abstracts, and (b) using versus not using the *SeparationCode*, *HospitalTransferFrom* and *HospitalTransferTo* variables. We did not evaluate a gap of zero days because hospital abstracts before 2004 only recorded dates without times, so that inter-hospital transfers using a gap of zero days would be misclassified if they began before midnight and ended after midnight. The rationale for allowing gaps up to two calendar days was to allow for a degree of occasional miscoding of hospital entry and/or separation dates.

We ignored abstract#2 if its admission and discharge dates were completely contained within those of abstract#1. This could occur if a patient was sent from hospital A to hospital B for a planned short time (e.g. a procedure), and anticipating return, the abstract in hospital A was not closed at the time of the transport to hospital B. Furthermore, when using the *HospitalTransferFrom* and *HospitalTransferTo* variables as part of identifying inter-hospital transfers, we tested only for the presence of codes representing transfer to or from any acute care hospital; not insisting on identification of the specific hospital was done to account for expected inaccuracy in the coding of specific hospitals. Five combination methods were used to create episodes of ICU-containing hospital care:

Method 1: No transfers. In this method, every hospital abstract was treated as though it was an entire hospital episode. This method intentionally ignored the possibility of transfers, and made no attempt to combine abstracts into episodes, no matter how brief the gap between them. Accordingly, it should over-estimate the number of episodes of care and under-estimate episode LOS, though the degree of inaccuracy is not known. Method 1 served as a baseline for comparison with the other methods, which attempted to identify inter-hospital transfers and combine corresponding abstracts.

Method 2: Allow a ≤1-day gap, and require an indication of inter-hospital transfer. Two hospital abstracts with a gap of ≤1 calendar day were considered part of the same hospital episode only if some indication of patient transfer to or from another hospital was also present, either: (A) the *SeparationCode* or *HospitalTransferTo* variable in abstract#1 indicated transfer to another hospital, or (B) the *HospitalTransferFrom* variable in abstract#2 indicated transfer from another hospital

Method 3: Allow a ≤1-day gap, with no requirement for an indication of inter-hospital transfer. Hospital abstracts with a gap of ≤1 calendar day were considered part of the same hospital episode.

Method 4: Allow a ≤2-day gap, and require an indication of inter-hospital transfer. Similar to Method 2 above, but the abstracts could be separated by up to two calendar days.

Method 5: Allow a ≤2-day gap, with no requirement for an indication of inter-hospital transfer. Similar to Method 3 above, but the abstracts could be separated by up to two calendar days. Method 5 should allow the most combinations of all the methods used, generating the lowest number of episodes of care, and the longest LOS.

The result for each patient was one or more hospital episodes, each constructed from one or multiple hospital abstracts. From these, the hospital episodes containing at least one ICU record (ICU-containing hospital episodes) were retained for subsequent construction of ICU episodes. The LOS of hospital episodes including multiple abstracts was calculated as the elapsed time from the beginning of the initial abstract to the end of the final abstract.

### Identifying episodes of ICU care

With ICU-containing hospital episodes identified, those that included only a single ICU record necessarily contained just one ICU episode. Hospital episodes including multiple ICU records could contain one or more ICU episodes, dependent on the presence of inter-ICU transfers. However, any method to identify inter-ICU transfer had to accommodate: (i) the possibility of inter-ICU transfer with a substantial delay, such as an intervening surgery, or transport between ICUs in remote hospitals, (ii) occasional inaccuracy of recorded dates/times, (iii) temporary transfer to an ICU in another hospital (e.g., to perform specific procedures) while the ICU bed in the sending hospital is retained for the patient’s planned return, and (iv) the fact that ICU readmissions sometimes occured after only a brief time on a ward.

The data elements we considered for identifying inter-ICU transfers were the timing of ICU entry and separation, and patient location before and after ICU admission. Unfortunately, pre-ICU and post-ICU location information is problematic in both sources of ICU records. While the WICUDB contains fields for pre-ICU and post-ICU locations, these are only reliable if they are within the six hospitals included in the WICUDB. And as the ICU records for the other 10 provincial do not contain explicit information about the pre-ICU and post-ICU locations. Because of this limitation, the sole criterion used for combining successive ICU records contained within a hospital episode was the time gap between ICU records.

Five methods were used for assessing the number and length of ICU episodes. These were applied in parallel with the methods used for hospital episodes. In Method 1 every ICU record was taken to represent an entire ICU episode. The other methods assessed ICU gaps of ≤1-day or ≤2-days. When a ≤1-day gap was used for adjacent hospital abstracts in Methods 2 and 3, the same gap was allowed between adjacent ICU records; and similarly for the ≤2-day gap in Methods 4 and 5. As above, gaps between ICU records of up to two calendar days allowed for combining records in the presence of a degree of occasional miscoding of ICU entry and/or separation timing. The LOS of ICU episodes including multiple records was calculated as the elapsed time from the beginning of the initial record to the end of the final record.

## Results

During the 8 year study period, 41,181 distinct Manitoba residents received ICU care in Manitoba hospitals. This care was identified in 48,551 hospital abstracts, and 53,246 ICU records, reflecting the fact that some individuals were hospitalized more than once, and that some hospital stays involve more than one episode of ICU care. If no attempt was made to combine successive records (Method 1, Table 1), these were also the number of hospital and ICU episodes. However, these numbers ignore patient transfers between hospitals and/or ICUs.

**Table 1:** Number of hospital and ICU episodes of care, by combination method

	**No combining of abstracts or records**	**≤ 1 day gaps**		**≤ 2 day gaps**	
		**Indication of inter-hospital transfer**		**Indication of inter-hospital transfer**	
		**Used**	**Not used**	**Used**	**Not used**
	**Method 1**	**Method 2**	**Method 3**	**Method 4**	**Method 5**
***Number of episodes***					
Hospital episodes	48,551	45,226	45,113	45,221	45,019
ICU episodes	53,246	48,312	48,290	47,777	47,716
***Difference*vs.*Method 1***					
Hospital episodes	--	6.8%	7.1%	6.9%	7.3%
ICU episodes	--	9.3%	9.3%	10.3%	10.4%

Table 1 shows how the different combination methods influenced the number of episodes of care. The main finding is that compared to any of the four algorithms used to identify patient transfers (Methods 2–5), ignoring the possibility of patient transfers (Method 1) resulted in 7-10% overestimation of the number of episodes of care. In contrast, there were only small differences between combination Methods 2–5. The number of episodes allowing for gaps of up to two days were very slightly lower than for ≤ 1 day gaps (Method 4 vs. Method 2; Method 5 vs. Method 3); amounting to differences of 0.01–0.2% for hospital episodes and 1.1-1.2% for ICU episodes. And finally, the number of both hospital and ICU episodes differed by ≤ 0.5% according to whether or not we required an additional indication of inter-hospital transfer (Method 2 vs. Method 3; Method 4 vs. Method 5). As expected, Method 5, which allowed a 2-day time gap and did not require an indication of inter-hospital transfer, was the most permissive in identifying transfers, and resulted in the smallest number of episodes.

Analysis of lengths of hospital and ICU stay is shown in Table 2. The main finding is that ignoring patient transfers (Method 1) lead to a 9-13% underestimation in median hospital and ICU LOS, regardless of which of Methods 2–5 were used to identify transfers. The impact on the mean LOS values for hospital episodes differed by up to 31%, likely influenced by a small fraction of patients who experienced transfers and long hospital LOS after leaving the ICU. Whether 1-day or 2-day gaps were used, and whether indications of inter-hospital transfer were required, resulted in only small differences in the mean and median lengths-of-stay of hospital and ICU episodes.

**Table 2:** Lengths-of-stay in hospital and ICU, by combination method

	**No combining of abstracts or records**	**≤ 1 day gaps**		**≤ 2 day gaps**	
		**Indication of inter-hospital transfer**		**Indication of inter-hospital transfer**	
		**Used**	**Not used**	**Used**	**Not used**
	**Method 1**	**Method 2**	**Method 3**	**Method 4**	**Method 5**
***Hospital LOS (days)***					
Mean ± SD	16.7 ± 33.6	21.5 ± 41.7	21.9 ± 43.8	21.5 ± 41.7	21.9 ± 42.1
Difference vs. Method 1	--	28.7%	31.1%	28.7%	31.1%
Median (IQR)	8 (4–16)	9 (4–21)	9 (5–21)	9 (4–21)	9 (5–21)
Difference vs. Method 1	--	12.5%	12.5%	12.5%	12.5%
***ICU LOS (hours)***					
Mean ± SD	89.1 ± 141.8	98.8 ± 172.7	99.0 ± 173.0	100.3 ± 175.1	100.5 ± 175.3
Difference vs. Method 1	--	10.9%	11.1%	12.8%	12.9%
Median (IQR)	48.8 (24.0-97.3)	53.3 (24.0-106.3)	53.3 (24.0-106.5)	54.2 (24.0-108.7)	54.3 (24.0-108.9)
Difference vs. Method 1	--	9.2%	9.2%	11.1%	11.3%

*LOS*, length-of-stay; *SD*, standard deviation; *IQR*, interquartile range.

Tables 3 and 4 show, respectively, the number of hospital abstracts and ICU records that were combined into hospital/ICU episodes using combination Method 5. The distributions for combination Methods 2–4 were similar. In 75% of hospital episodes and 90% of ICU episodes, the episodes were comprised of a single abstract or record. This indicates the presence of inter-hospital transfers in 25% of ICU-containing hospital episodes, and inter-ICU transfers in 10% of ICU episodes; the majority of such episodes included two hospital abstracts or ICU records.

**Table 3:** Distribution of the number of hospital abstracts per ICU-containing hospital episode, for combination Method 5

**# Hospital abstracts per hospital episode**	**# of hospital episodes**	**% of hospital episodes**
1	33,548	74.5
2	7,801	17.3
3	2,905	6.5
4	493	1.1
5	179	0.4
6	63	0.14
7	16	0.04
8 or more	14	0.02

**Table 4:** Distribution of the number of ICU records per ICU episode, for combination Method 5

**# ICU records per ICU episode**	**# of ICU episodes**	**% of ICU episodes**
1	43,054	90.2
2	3,861	8.1
3	670	1.4
4	111	0.2
5 or more	20	0.04

## Discussion

Inpatient databases in Manitoba, like most other places, create separate entries for each time an individual enters a hospital or ICU. In the presence of inter-hospital or inter-ICU transfers, considering each entry to be a separate episode of care is incorrect. Patients may be transferred for a number of reasons, including accessing medical services unavailable at the original location, or as part of integrated bed management in health systems with multiple hospitals. Both occur in Manitoba, and we found that inter-hospital and inter-ICU transfers are common for ICU patients in this province. The consequences of failing to account for such transfers include inaccurate assessment of important parameters such as admission rates, readmission rates, mortality, and lengths-of-stay. However, erroneously combining entries that represent separate episodes of care will also produce incorrect values.

Our study quantifies some of the inaccuracies that result from failing to take account of transfers among these patients. Doing so overestimated the number of episodes by 7-10%, while concomitantly underestimating mean or median lengths-of-stay by 9-30%. Our study also shows that the details of the combination method used, at least among the options considered here, made relatively little difference. The number of episodes and LOS differed surprisingly little whether the maximum allowed gap between successive entries was one or two calendar days. These parameters also were insensitive to whether or not another indication of inter-hospital transfer was used in addition to the time gap criterion. Of course, it seems wise to include information regarding transfers when available, as they likely reduce erroneous combining of entries which are actually separate episodes of care.

Although published studies of inpatient care have used simple combination rules [5], an extensive search of published literature identified no publications assessing methods for constructing episodes of inpatient care from the individual entries that comprise most databases. This raises the concern of whether some of the database-derived literature on inpatient care overestimated admission rates and readmission rates, while underestimating lengths-of-stay and mortality rates. Of course, we would expect such difficulties to be of practical magnidue only where substantial fractions of hospitalized patients undergo inter-hospital or inter-ICU transfers, as occurs in Manitoba.

The main strengths of our work are that it used population-based data from an entire Canadian province, over a substantial time period, and that it demonstrated the impact of several different methods of identifying the presence of patient transfers. It also has several limitations. First, it only included persons whose hospital care included admission to an ICU. Therefore, the performance of the combination methods for identifying hospital episodes assessed here might be different in different patient groups, such as unselected hospitalized patients. Second, we did not assess other relevant outcomes, including rates of admission, readmission, and mortality. Third, our data do not allow us to identify the “true” situation. The gold standard for identifying episodes of hospital and ICU care, manual review of all inpatient charts in the province, is impractical. However, the high degree of similarity between the results of Methods 2–5 suggests that they are unlikely to be very divergent from that truth. Fourth, we did not evaluate time gaps between hospital abstracts or ICU records exceeding two calendar days. However, data showing that ICU readmission after longer time periods are more likely to be for different reasons than the initial admission [6], implies that allowing longer gaps would increase the rate of misclassifying readmissions as being part of a single episode of care.

## Conclusions

We demonstrated that failing to account for inter-hospital and inter-ICU transfers resulted in inaccurate assessment of parameters relevant to researchers, clinicians, and policy-makers. The four methods we evaluated for identifying transfers resulted in similar estimates of the number and length of episodes. This implies that, within limits, the method chosen for identifying transfers is less critical than the step of simply not treating every entry as an independent episode. In addition, we showed that these methods for identifying episodes of ICU and hospital care can be implemented in a large, complex dataset.

## Competing interests

The authors declare that they have no competing interests.

## Authors’ contributions

RF and AG conceived the study. RF, AG, KO, CR and MY participated in the design of the study. MY and KM participated in coordination of the study. MY performed the statistical analysis. RF and AG drafted the manuscript. All authors read the draft, provided feedback, and approved the final manuscript.

## Pre-publication history

The pre-publication history for this paper can be accessed here:

http://www.biomedcentral.com/1471-2288/12/133/prepub
